# Genetic Variation in Glutathione-Related Genes and Body Burden of Methylmercury

**DOI:** 10.1289/ehp.10804

**Published:** 2008-02-14

**Authors:** Karin Schläwicke Engström, Ulf Strömberg, Thomas Lundh, Ingegerd Johansson, Bengt Vessby, Göran Hallmans, Staffan Skerfving, Karin Broberg

**Affiliations:** 1 Department of Occupational and Environmental Medicine, Lund University Hospital, Lund, Sweden; 2 Department of Odontology, Faculty of Medicine, Umeå University, Umeå, Sweden; 3 Department of Public Health and Caring Sciences, Uppsala University, Uppsala, Sweden; 4 Department of Public Health and Clinical Medicine, Umeå University, Umeå, Sweden

**Keywords:** *GCLC*, *GCLM*, *GSTP1*, metabolism, methylmercury, polymorphism

## Abstract

**Background:**

Exposure to toxic methylmercury (MeHg) through fish consumption is a large problem worldwide, and it has led to governmental recommendations of reduced fish consumption and blacklisting of mercury-contaminated fish. The elimination kinetics of MeHg varies greatly among individuals. Knowledge about the reasons for such variation is of importance for improving the risk assessment for MeHg. One possible explanation is hereditary differences in MeHg metabolism. MeHg is eliminated from the body as a glutathione (GSH) conjugate.

**Objectives:**

We conducted this study to assess the influence of polymorphisms in GSH-synthesizing [glutamyl-cysteine ligase modifier subunit (*GCLM*-588) and glutamyl-cysteine ligase catalytic subunit (*GCLC*-129)] or GSH-conjugating [glutathione *S*-transferase pi 1 (*GSTP1*–105 and *GSTP1*–114)] genes on MeHg retention.

**Methods:**

Based on information obtained from questionnaires, 292 subjects from northern Sweden had a high consumption of fish (lean/fat fish two to three times per week or more). We measured total Hg in erythrocytes (Ery-Hg) and long-chain n-3 polyunsaturated fatty acids in plasma (P-PUFA; an exposure marker for fish intake).

**Results:**

The *GSTP1* genotype modified Ery-Hg; effects were seen for *GSTP1*–105 and −114 separately, and combining them resulted in stronger effects. We found evidence of effect modification: individuals with zero or one variant allele demonstrated a steeper regression slope for Ery-Hg (*p* = 0.038) compared with individuals with two or more variant alleles. The *GCLM*-588 genotype also influenced Ery-Hg (*p* = 0.035): Individuals with the *GCLM*-588 TT genotype demonstrated the highest Ery-Hg, but we saw no evidence of effect modification with increasing P-PUFA.

**Conclusions:**

These results suggest a role of GSH-related polymorphisms in MeHg metabolism.

Methylmercury (MeHg) is a very reactive organometal compound. Exposure especially affects the nervous system. Hence, low-level exposure during pregnancy may cause impaired development in infants and children (National Research Council 2000). In most populations, fish is the major source of MeHg (International Programme on Chemical Safety 1990). The elimination half-time of MeHg varies greatly among individuals, ranging from 45 to 70 days (Clarkson 2002), but extreme values, up to almost 190 days, have been reported (Al-Shahristani and Shihab 1974). One explanation of this variation might be hereditary differences in MeHg metabolism.

MeHg is eliminated in the bile as a glutathione (GSH) conjugate (Ballatori and Clarkson 1985; Dutczak and Ballatori 1994), and polymorphisms in GSH-synthesizing or GSH-conjugating enzymes may thus influence the elimination capacity. The rate-limiting enzyme for GSH synthesis is glutamyl-cysteine ligase (GCL), which is composed of a catalytic subunit [GCLC; Unigene accession no. Hs.654465; National Center for Biotechnology Information (NCBI) Unigene Database 2006b] and a modifier subunit (GCLM; Unigene accession no. Hs.315562). Polymorphisms affecting GSH production (lower promoter activity for the variant alleles) have been found in both subunits: a C→T nucleotide substitution at position −129 in *GCLC* (*GCLC*-129) (Koide et al. 2003) and a C→T nucleotide substitution at position −588 in *GCLM* (Nakamura et al. 2002).

The glutathione *S*-transferases (GSTs), which conjugate GSH to a wide variety of electrophilic compounds (Hayes and Strange 2000; Strange and Fryer 1999), may also affect the metabolism of MeHg. The GST family comprises several genes, many of which are polymorphic in humans. Glutathione *S*-transferase pi 1 (*GSTP1*; Unigene accession no. Hs.523836) exhibits a number of variations, of which the Ile105Val and Ala114Val amino acid substitutions both are associated with differences in enzyme activity and substrate preferences (Ali-Osman et al. 1997; Sundberg et al. 1998; Zimniak et al. 1994).

In a recent study by our group (Custodio et al. 2005), individuals with one variant allele for either *GCLC*-129 or *GSTP1*-114 were found to have a higher retention of mercury in erythrocytes (Ery-Hg) compared with individuals with other genotypes but similar exposure [level of long-chain n-3 polyunsaturated fatty acids in plasma (P-PUFA) was used as a proxy for fish consumption/MeHg exposure]. A limitation of that study was that most subjects had low levels of fish consumption, and thereby low Ery-Hg. The aim for this study was to further investigate the associations between Ery-Hg and GSH-related genotypes at higher MeHg exposures. For this purpose, we chose polymorphisms in the three genes, for which effects on Hg retention have been seen for either MeHg (*GCLC* and *GSTP1*) or inorganic Hg (*GCLM*) (Custodio et al. 2004).

## Materials and Methods

### Study population

The study subjects were selected from the Västerbotten Intervention Program (VIP) cohort. VIP is a long-term project intended for health promotion of the population of Västerbotten, a county in northern Sweden. Since 1985, all individuals at 40, 50, and 60 years of age in the population of the county were invited for screening, and the cohort included 94,630 sampling occasions from 74,690 unique individuals by June 2006. Blood samples were taken from the VIP cohorts in a uniform way after an overnight fasting period, and the samples were stored as plasma, erythrocytes, or buffy coat at −80°C. The survey procedures are detailed elsewhere (Hallmans et al. 2003). The study subjects filled out a health/lifestyle questionnaire (Johansson et al. 2002), and a random sample of 300 individuals was chosen from among individuals consuming fish at least 2.5 times a week (2,648 individuals in total). The individuals were matched to our previous study for age and sex. Among these, we had erythrocytes and DNA samples for 292 individuals. The mean age was 49 years, with a range of 29–61 year. Of this group, 207 individuals (71%) were male and 85 (29%) were female. The samples were collected between 1992 and 2002. The study was approved by the Regional Ethics Committee of Umeå University.

We complied with all applicable requirements of the United States and international regulations (including institutional review board approval). Participants gave written informed consent before the study.

### Measurement of Ery-Hg and P-PUFA

We determined Ery-Hg in acid-digested samples using cold vapor atomic fluorescence spectrometry (Sandborgh-Englund et al. 1998). The limit of detection, calculated as 3 × SD for the blanks, was 0.10 μg/L. All samples were prepared in duplicate, and the method imprecision (calculated as the coefficient of variation for duplicate preparations measurements) was 3.7%. The analytical accuracy was checked against reference material, consisting of certified human blood samples (lot M0401 and M0407) obtained from the Centre de Toxicologie du Quebec, International Comparison Program, Quebec, Quebec, Canada). The results obtained were 1.8 ± 0.10 μg/L (mean ± SD; lot M0401) and 9.2 ± 0.26 μg/L (lot M0407) versus certified 2.0 and 9.4 μg/L, respectively.

Plasma fatty acids were separated by gas–liquid chromatography after separation of the lipids by thin-layer chromatography and transmethylation, as described elsewhere (Boberg et al. 1985). The relative amounts of the fatty acids were expressed as a percentage of all fatty acids analyzed. We calculated P-PUFA as the fraction of (%) eicosapen-taenoic acid (20:5 n-3) and docosahexaenoic acid (22:6 n-3) in plasma phospholipids, which are closely associated with fish intake (Hallgren et al. 2001; Svensson et al. 1993).

### Genotype analyses

We used the Taqman allelic discrimination assay (ABI 7000; Applied Biosystems, Foster City, CA, USA) to analyze separately all four single nucleotide polymorphisms (SNPs): *GCLC*-129, *GCLM*-588, *GSTP1*-105, and *GSTP1*-114. The primers and probes have been described previously (Custodio et al. 2005). Each real-time polymerase chain reaction (PCR) assay (except for *GSTP1*-114) was performed with a reaction volume of 25 μL containing 1× Universal Taqman mix (Applied Biosystems), 5 ng DNA, 0.9 μM of each primer, and 0.2 μM of each probe, with the following program: 50°C for 2 min and 95°C for 10 min, followed by 40 cycles of 95°C for 15 sec and 60°C for 1 min. The *GSTP1*-114 reaction contained 1 U *Taq* polymerase (*Taq* Platinum; Invitrogen, Carlsbad, CA, USA), 1× PCR buffer, 2.5 mM MgCl_2_, 0.8 mM dNTP (Amersham Biosciences, Piscataway, NJ, USA), 1× ROX (6-carboxyl-X-rhodamine; Invitrogen), 2 ng DNA, 1 μM of each primer, 0.08 μM of each probe, and double-distilled water in a total volume of 25 μL, and followed the program described above. We included controls for each genotype in each run, and genotyping was repeated on 5% of the samples. There was a perfect agreement between the original and repeat genotyping runs in all of the four SNPs analyzed.

### Statistical analysis

We tested deviations from Hardy-Weinberg equilibrium using the Fisher-Freeman-Halton test (StatXact; Cytel Inc., Cambridge, MA, USA). We analyzed Spearman’s correlation (*r_s_*) to evaluate the “strongest” exposure marker of MeHg (P-PUFA, total fish intake, fat fish intake, or lean fish intake). To explore effects at different levels of exposure, subjects were divided into three groups of equal sizes, according to the strongest exposure marker variable. Analysis of variance (ANOVA) techniques were employed to analyze, within the different exposure groups, the effects of genotype (single gene effects, as well as combined effects of the two *GSTP1* polymorphisms) on natural log (ln)–transformed Ery-Hg, as the dependent variable. Because of the low number of individuals with the homozygote variant genotype, *GSTP1*-114 AlaVal and ValVal were pooled (AlaVal + ValVal), as well as *GCLC*-129 CT and TT (CT + TT). For the multivariate analyses, we used only the “strongest” exposure marker of MeHg—as in the ANOVAs (the different exposure marker variables were highly correlated)—as a numerical (not categorized) independent variable, with ln Ery-Hg as the dependent variable. We carefully evaluated the form of association between the exposure marker and ln Ery-Hg variables.

Other potentially influential independent variables (age, sex, alcohol consumption, snuff usage, year of sampling) were included in the model if they had a *p*-value < 0.2. Variables that did not influence the results were thereafter excluded from the model (i.e., sex, *p* = 0.21; alcohol consumption, *p* = 0.31; and snuffing, *p* = 0.78). We used a multivariate model with an interaction term between exposure and genotype to account for multiplicative effect modification. If no effect modification was present, we used a model without the interaction term.

We also analyzed effects of dual-gene polymorphisms, where genotype was defined as the total number of variant alleles for both genes. The effects of the genes were first assessed separately for each genotype; genotypes were subsequently grouped based on the effect estimates. We used data from individuals with the most variant alleles as references values. We performed post hoc tests using the least significant difference method to account for comparisonwise error rate. All statistical analyses were performed using SPSS software (version 14; SPSS, Chicago, IL, USA).

## Results

### Exposure markers

Summary data for Ery-Hg, P-PUFA, and fish consumption (total, fat, and lean fish intake) are presented in Table 1. For fish consumption, the correlations with Ery-Hg were as follows: total fish, *r_s_* = 0.078, *p* = 0.18; fat fish, *r_s_* = 0.17, *p* = 0.004; and lean fish, *r_s_* = −0.12, *p* = 0.036.

**Figure 1:**
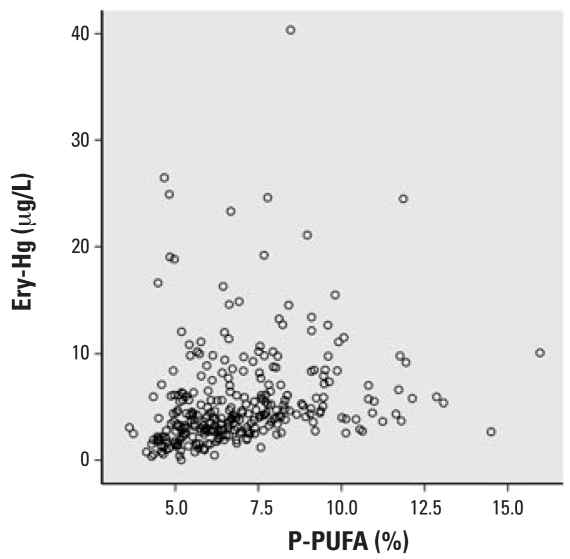
Relationship between P-PUFA and Ery-Hg.

**Figure 2:**
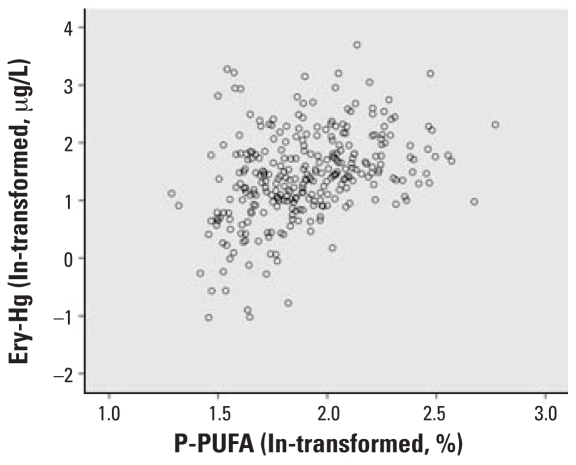
Relationship between P-PUFA (ln transformed) and Ery-Hg (ln transformed).

**Figure 3:**
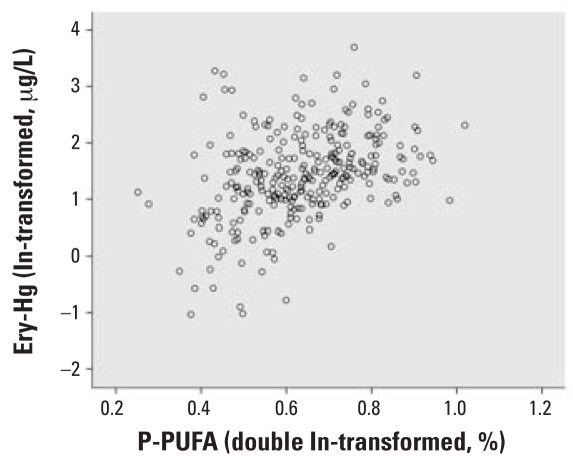
Relationship between P-PUFA (double ln transformed) and Ery-Hg (ln transformed).

P-PUFA had the strongest correlation with Ery-Hg (*r_s_* = 0.41, *p* < 0.001; Figure 1) and was thus chosen as the exposure marker. The exposure markers were strongly correlated with each other: P-PUFA was positively correlated with total fish intake (*r_s_* = 0.16, *p* = 0.005) and fat fish intake (*r_s_* = 0.30, *p* < 0.001), but it was negatively correlated with lean fish intake (*r_s_* = −0.20, *p* < 0.001). Hence, P-PUFA was employed as the exposure marker variable. In the multivariate analyses, P-PUFA was double log transformed to fit into linearity, which is more appropriate for the general linear model used by SPSS. Figures 2 and 3 clarify the changes in linearity by P-PUFA transformations. Two more covariates were included in the multivariate analysis: age (*r_s_* = 0.17, *p* < 0.003) and year of sampling (*r_s_* = −0.16, *p* < 0.004). We found a time trend with decreasing Ery-Hg for each year of sampling (but no similar annual decrease in P-PUFA; data not shown).

### Effect of genotype on Ery-Hg: ANOVA

The genotype frequencies are presented in Table 2. All genes were in Hardy-Weinberg equilibrium, except for *GCLM*-588 (*p* < 0.001). ANOVA results are presented in Table 3.

### Glutathione-synthesizing genes (*GCLM* and *GCLC*)

No significant results were seen in the ANOVA results.

### GSTP1

Ery-Hg was affected by genotype only in the highest exposure group (*p* = 0.050), where individuals with the ValVal allele for *GSTP1*-105 had lower Ery-Hg compared with individuals with IleIle (*p* = 0.046) and IleVal (*p* = 0.057) genotypes. Individuals carrying at least one Val allele for *GSTP1*-114 had lower Ery-Hg compared with individuals with the AlaAla genotype (*p* = 0.014).

### SNP combinations

Individuals with two or more variant alleles of either *GSTP1*-105 or *GSTP1*-114 had lower Ery-Hg than did individuals with zero (*p* = 0.004) or one (*p* = 0.071) variant allele. For all individuals with one variant allele in the *GSTP1*-105 and *GSTP1*-114 combination, this allele was *GSTP1*-105Val.

### Effect of genotype on Ery-Hg: multivariate analysis

The final model was as follows: ln Ery-Hg = intercept + β_1_ × genotype + β_2_ × age + β_3_ × year of sampling + β_4_ × P-PUFA + β_5_ × (genotype × P-PUFA). If no effect modification was present, a model without interaction term was performed: ln Ery-Hg = intercept + β_1_ × genotype + β_2_ × age + β_3_ × year of sampling + β_4_ × P-PUFA.

### Glutathione-synthesizing genes (*GCLM* and *GCLC*)

**Figure 4:**
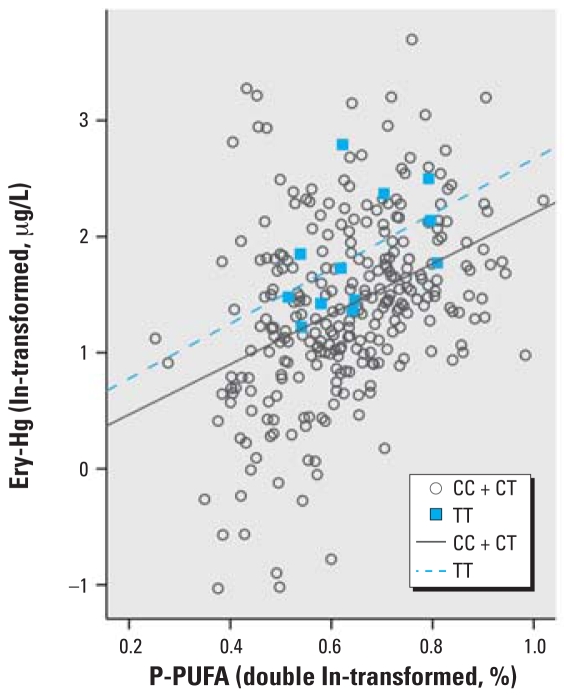
Ery-Hg as a function of P-PUFA for different *GCLM* C588T genotypes. The regression lines do not reflect adjustments for age and year of sampling, as does the multivariate regression model presented in the text.

No effects were seen in the model with an interaction term (Table 4). Hence, we analyzed the data in a model without an interaction term, where *GCLM* demonstrated significant effects on Ery-Hg (*p* = 0.035) (Figure 4); TT carriers had higher Ery-Hg than did the other genotypes. Geometric means adjusted for P-PUFA, age, and year of sampling were as follows: for *GCLM*-588, CC, 4.3 μL/g; CT, 3.4 μL/g; and TT, 5.5 μL/g, yielding significant associations for CT versus TT (*p* = 0.032) and CT versus CC (*p* = 0.031). The association between CC versus TT carriers was nonsignificant (*p* = 0.22). No significant relationships were seen for *GCLC* (data not shown).

### GSTP1

Effect modifications were seen for *GSTP1*-105 and *GSTP1*-114 (Table 4). For *GSTP1*-105, individuals with the Ile allele present (i.e., zero or one of the variant Val allele) demonstrated a steeper regression slope for Ery-Hg. The AlaAla carriers for *GSTP1*-114 followed the same pattern as the individuals with *GSTP1*-105 Ile allele present.

### SNP combinations

**Figure 5:**
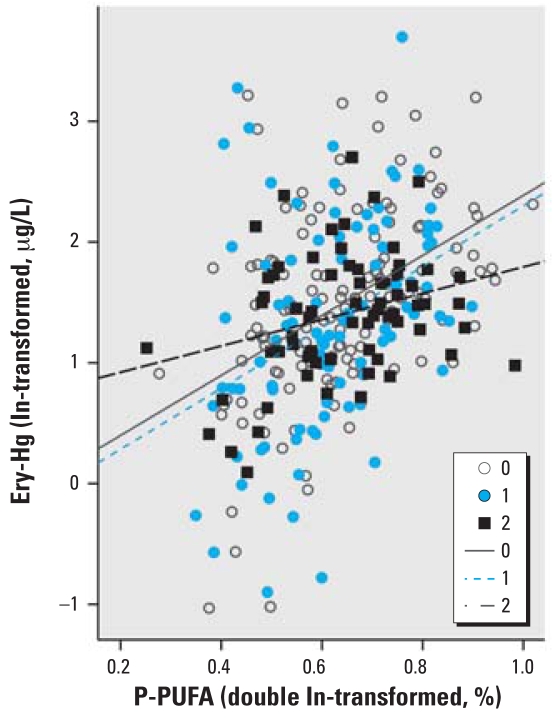
Ery-Hg as a function of P-PUFA for different numbers of variant alleles for *GSTP1* Ile105Val and *GSTP1* Ala114Val. The regression lines are aimed at illustrating the general direction of interaction; thus, they were not derived from the multivariate regression model presented in Table 4.

The dual-polymorphism effects for *GSTP1*-105 and -114 were stronger than for each gene separately (for all individuals with one variant allele, this allele was *GSTP1*-105Val) (Figure 5, Table 4). An effect modification was indicated for the combination of *GCLC*-129 and *GSTP1*-114 (Table 4), where subjects with zero or one variant allele demonstrated a steeper regression slope compared with individuals with two variant alleles. A similar effect was also seen for the combination of *GCLC*-129 and *GSTP1*-105 (Table 4).

## Discussion

Results of the present study indicate that polymorphisms in *GCLM* and *GST* genes modify the relationship between exposure to and retention of MeHg.

Genetic influences on metal metabolism have been established for only a few toxic metals. *ALAD* (δ-aminolevulinic acid dehy-dratase) genotype and lead exposure is the most studied interaction; at high levels of exposure and in comparison with *ALAD*-1 subjects, heterozygous or homozygous *ALAD*-2 carriers demonstrate increased blood lead levels (Kelada et al. 2001; Scinicariello et al. 2007). The *VDR* (vitamin D receptor) genotype has also been shown to influence the level of blood lead. Thus, Haynes et al. (2003) showed that children with the Ff genotype displayed lower blood lead levels than did those with the FF genotype. For exposure to inorganic arsenic, the *AS3MT* genotype appears to have a strong influence on the fraction of the most toxic metabolite, monomethylated arsenic, in urine (Meza et al. 2005; Schläwicke Engström et al. 2007). For most other metals, gene–environment interactions are largely unknown, and MeHg is no exception. Custodio et al. (2005) reported that individuals with one variant allele for either *GCLC*-129 or *GSTP1*-114 had higher Ery-Hg compared with individuals with other genotypes but similar exposure. To our knowledge, no other studies regarding gene–exposure interactions for MeHg have been performed.

There are several methodologic issues to take into consideration. Obviously, evaluating the modification of the relationship between exposure and retention of MeHg by genes requires accurate estimates of both. Hence, the first methodologic issue is the choice of an appropriate exposure marker for MeHg. The exposure to MeHg depends on both the intake of fish and the MeHg concentration in the fish. Because we could not measure MeHg in fish, we had to rely on a proxy. A way to assess the usefulness of the different options is to look for correlations with Ery-Hg. The association between P-PUFA and Ery-Hg was much stronger than the association between Ery-Hg and the self-reported variables—total, fat, and lean fish intake—in accordance with previous studies (Custodio et al. 2004; Hallgren et al. 2001), which probably reflects the fact that the number of fish meals reported is a poor measure of the amount of fish consumed. Hence, we used P-PUFA as an exposure marker, fully aware of the fact that P-PUFA is a very imprecise marker. However, this would bias toward the null. To achieve linearity, P-PUFA was double ln transformed. However, the difference in results between ln %PUFA and double ln %PUFA is not that large. Some examples on this (ln-transformed PUFA vs. double ln-transformed PUFA): *GSTP1*-105, 0.090 versus 0.077; *GSTP1*-114, 0.054 versus 0.061; and combination *GSTP1*-105 and *GSTP1*-114, 0.030 vs. 0.038.

We found a time trend of decreasing Ery-Hg. Another study from the same geographic area also shows that Ery-Hg is declining, with an average annual decrease of 5.8% between the years 1990 and 1999 (Wennberg et al. 2006). Ery-Hg rose with increasing age in our study. This finding is probably explained by more than just higher fish consumption among elderly; the correlation between age and Ery-Hg remained after adjustments for P-PUFA (data not shown). Increasing age may also be associated with impairment of the metabolism of MeHg. Indeed, both GSH and GST levels have been shown to decrease with age (Voss and Siems 2006).

The second issue is the use of Ery-Hg as a marker for MeHg retention. Inorganic Hg can, to some extent, contribute to the levels of Ery-Hg. However, Ery-Hg is mainly MeHg and is a good marker of MeHg body burden (Berglund et al. 2005; International Programme on Chemical Safety 1990). Part of the inorganic Hg could be attributed to demethylation of MeHg during sample treatment and analysis (Bergdahl et al. 1995; Berglund et al. 2005). Additionally, the major source of inorganic Hg, dental amalgam, has decreased significantly in Sweden (National Swedish Board of Social Welfare 2002; Sundberg et al. 2000), including in the present region (Wännman et al. 2004).

The third issue regards Hardy-Weinberg disequilibrium for *GCLM*-588. The allele frequency reported here is similar to another study on Danish and Swiss populations where equilibrium was reported (Tosic et al. 2006). The preceding gene–environment study for MeHg with individuals from the same geographic area (Custodio et al. 2005) also showed linkage disequilibrium, whereas in other studies by our group, using the same methods but in other populations, we observed equilibrium (Custodio et al. 2004; Jönsson et al. 2007). This may indicate that the linkage disequilibrium is population specific.

The fourth issue concerns multiple testing. Several genotypes have been analyzed, which increases the possibility of false-positive findings. A false-positive report probability depends on the subjective prior belief regarding a true association (Wacholder et al. 2004). We did not adjust for multiple effect estimation, based on multiple models for different genotypes. However, we performed post hoc testing (with adjustments for multiple testing) when comparing multiple subgroups (genotypes) of a given SNP. Further, some results should be cautiously interpreted because they were based on comparisons in groups with small number of individuals (e.g., the ANOVA results, dividing the study subjects in three exposure groups).

The present study indicates that the individual differences in retention and elimination of MeHg are partly due to hereditary differences in genes conjugating and synthesizing GSH. Individuals with two variant alleles of the *GCLM*-588 polymorphism (TT) had higher Ery-Hg, but there was no evidence of effect modification. The *GCLM*-588T allele is associated with lower promoter activity and lower levels of GSH in plasma (Nakamura et al. 2002). An effect of the T allele genotype (CT + TT), giving increased levels of Hg in blood, plasma, and urine, has been seen for inorganic Hg (Custodio et al. 2004), which is also metabolized by conjugation to GSH. However, we saw similar effects only for the TT genotype, whereas individuals with the CT genotype had lower Ery-Hg than did those with either the TT or CC genotype. It appears that *GCLM* does not exhibit an allele–dose effect. A recessive model appears to be more plausible, where TT individuals have higher MeHg compared with carriers of the other genotypes. Still, this does not explain the lower levels of the CT genotype.

We found no results in the dual gene combinations that included the *GCLM*-588 polymorphism. This was probably because the individuals with the lowest and those with the highest Ery-Hg levels (i.e., CT and TT genotypes) often were classified in the same group.

The polymorphisms in *GSTP1* (Ile105Val and Ala114Val) separately appear to modify the levels of Ery-Hg for different levels of P-PUFA, and the combination of those polymorphisms demonstrated a stronger effect than did each polymorphism alone. Individuals with zero or one variant allele had a steeper regression slope compared with individuals with two variant alleles. This resulted in significantly higher Ery-Hg at high exposures, which can be seen in the ANOVA results. No significant effects were seen at the low-exposure groups for any polymorphism. The functional impact of these variants on MeHg remains to be clarified. However, both amino acid exchanges are in the active site of the enzyme and influence the activity toward different substrates (Ali-Osman et al. 1997; Sundberg et al. 1998; Zimniak et al. 1994).

In the study by Custodio et al. (2005), individuals with one variant allele for either *GCLC*-129 or *GSTP1*-114 had a steeper regression slope, compared with those with zero variant alleles. In the present study, an effect was seen mainly among carriers with two variant alleles, but carriers with one allele demonstrated a nonsignificantly shallower (*p* = 0.35) regression slope compared with carriers with zero variant alleles. The individuals in the study of Custodio et al. (2005) had a lower exposure compared with the individuals in our study, which may explain some of the differences between the studies. The effect of the genotypes may differ according to the exposure. Moreover, the genetic effects are difficult to study at low exposure levels, and we cannot rule out the possibility that the finding from the first study may be a false-positive finding.

## Conclusion

Data from the present study indicate that hereditary factors influence the metabolism of MeHg. Two polymorphisms in *GSTP1* modified the Ery-Hg levels at high MeHg exposures, whereas homozygotes for the *GCLM*-588 polymorphism had higher Ery-Hg levels but showed no evidence of effect modification. However, the mode of inheritance for the *GCLM* polymorphism is not clear and needs to be studied further. We would like to stress the importance of information on genetic impact on MeHg metabolism, because it may mean a difference in susceptibility to toxic effects, which may influence the risk assessment and thus the foundation of preventive actions.

## Correction

In the acknowledgments of the original manuscript published online, the authors left out the word “no” in their declaration of competing financial interests. Their declaration has been corrected here.

## Figures and Tables

**Table 1:** Summary Ery-Hg and exposure data for the study population (*n* = 292).

Characteristic	Mean	Median	Range
Ery-Hg (μg/L)	5.5	4.2	< LOD–40
P-PUFA (%)	6.9	6.6	3.6–16
Fish consumption (no. of meals per day)			
Total	0.52	0.44	0.36–2.0
Fat fish	0.22	0.14	0–1.0
Lean fish	0.30	0.36	0.040–1.0

LOD, limit of detection.

**Table 2:** Genotype frequencies for *GCLC*-129, *GCLM*-588, *GSTP1*-105, and *GSTP1*-114.

Gene	Polymorphism	Genotype	Frequency (%)
*GCLC*	−129C/T	CC	88
rs17883901		CT	11
		TT	0.3
*GCLM*	−588C/T	CC	76
rs41303970		CT	20
		TT	4
*GSTP1*	Ile105Val	Ile/Ile	45
rs1695		Ile/Val	47
		Val/Val	9
*GSTP1*	Ala114Val	Ala/Ala	80
rs1138272		Ala/Val	19
		Val/Val	1

The rs (refSNP) numbers are from the SNP Database (NCBI 2006a).

**Table 3:** Ery-Hg (geometric mean) for different genotypes among the different exposure groups.

	Low (3.6–5.8% P-PUFA)		Intermediate (5.9–7.5% P-PUFA)		High (7.6–16% P-PUFA)	
Genotype	Ery-Hg	No.	Ery-Hg	No.	Ery-Hg	No.
*GCLC*-129^a^						
CC	2.9	83	3.9	85	5.9	90
CT + TT	3.3	14	4.1	13	7.9	7
*GCLM*-588						
CC	3.2	74	4.0	72	6.0	75
CT	2.2	20	3.4	19	5.7	18
TT	4.6	3	5.8	5	9.0	4
*GSTP1*-105						
IleIle	3.1	45	4.1	41	7.0*	44
IleVal	2.7	43	3.6	47	5.6	46
ValVal	3.7	9	5.2	9	4.4	7
*GSTP1*-114 Val allele^b^						
AlaAla	2.9	80	4.0	77	6.5*	75
AlaVal, ValVal	3.1	16	3.9	21	4.7	22
*GSTP1*-105 and *GSTP1*-114						
No variant alleles	3.0	44	4.1	40	7.0*	44
One variant allele	2.7	33	3.6	33	6.1	28
Two variant alleles	3.2	19	4.2	24	4.6	25

a *GCLC* CT and TT are pooled because of the low number of individuals with the TT genotype (*n* = 1).

b *GSTP1* AlaVal and ValVal are pooled because of the low number of individuals with the ValVal genotype (*n* = 4).

* *p* < 0.05 within the exposure group.

**Table 4:** Multivariate regression model.^a^

Polymorphism	Effect modification β_5_^b^	*p*-Value
*GCLC*-129 (CC vs. CT)	0.67	0.40
*GCLM*-588 (CC + CT vs. TT)	0.25	0.90
*GSTP1*-105 (IleIle + IleVal vs. ValVal)	1.8	0.077
*GSTP1*-114^b^ (AlaAla vs. ValVal + AlaVal)	1.3	0.061
*GSTP1*-105 and *GSTP1*-114 combination		
0 and 1 variant allele (222) vs. 2 variant alleles (68)	1.5	0.038
*GCLC*-129 and *GSTP1*-105		
0 and 1 variant allele (249) vs. 2 variant alleles (42)	1.3	0.095
*GCLC*-129 and *GSTP1*-114		
0 and 1 variant allele (262) vs. 2 variant alleles (27)	1.8	0.091
*GCLC*-129 and *GCLM*-588		
0 and 1 variant allele (270) vs. 2 variant alleles (20)	−1.0	0.41
*GCLM*-588 and *GSTP1*-105		
0 and 1 variant allele (226) vs. 2 variant alleles (64)	0.33	0.65
*GCLM*-588 and *GSTP1*-114		
0 and 1 variant allele (262) vs. 2 variant alleles (27)	0.66	0.50

a Genotypes are dichotomized, and referents are denoted last. Ery-Hg is ln transformed, and P-PUFA is double ln transformed. Only the results of the effect modification (genotype*P-PUFA) on Ery-Hg levels are presented.

b The term β5 denotes the difference between the inclinations of the regression slopes for Ery-Hg on P-PUFA.
